# Efficacy Differences of First-line EGFR-TKIs Alone *vs* in Combination with Chemotherapy in Advanced Lung Adenocarcinoma Patients with Sensitive *EGFR* Mutation and Concomitant Non-*EGFR* Genetic Alterations

**DOI:** 10.3779/j.issn.1009-3419.2022.102.34

**Published:** 2022-09-20

**Authors:** Guowei ZHANG, Ruirui CHENG, Yuanyuan NIU, Huijuan WANG, Xiangtao YAN, Mina ZHANG, Xiaojuan ZHANG, Jinpo YANG, Chunhua WEI, Zhiyong MA

**Affiliations:** 1 Department of Internal Medicine, The First Affiliated Hospital of Zhengzhou University, Zhengzhou 450000, China; 2 Department of Respiratory Medicine, The First Affiliated Hospital of Zhengzhou University, Zhengzhou 450000, China

**Keywords:** Lung neoplasms, *EGFR* mutation, Concomitant genetic alteration, Targeted therapy, Chemotherapy

## Abstract

**Background and objective:**

Epidermal growth factor receptor (*EGFR*) mutations are often associated with non-*EGFR* genetic alterations, which may be a reason for the poor efficacy of EGFR tyrosine kinase inhibitors (TKIs). Here we conducted this study to explore whether EGFR-TKIs combined with chemotherapy would benefit advanced lung adenocarcinoma patients with both sensitive *EGFR* mutation and concomitant non-*EGFR* genetic alterations.

**Materials and methods:**

Cases of advanced lung adenocarcinoma with *EGFR* mutation combined with concomitant non-*EGFR* genetic alterations were retrospectively collected. And the patients were required to receive first-line EGFR-TKIs and chemotherapy combination or EGFR-TKIs monotherapy. Demographic, clinical and pathological data were collected, and the electronic imaging data were retrieved to evaluate the efficacy and time of disease progression. Survival data were obtained through face-to-face or telephone follow-up. The differences between the two groups in objective response rate (ORR), disease control rate (DCR), progression-free survival (PFS) and overall survival (OS) were investigated.

**Results:**

107 patients were included, including 63 cases in the combination group and 44 cases in the monotherapy group. The ORR were 78% and 50% (*P*=0.003), and DCR were 97% and 77% (*P*=0.002), respectively. At a median follow-up of 13.7 mon, a PFS event occurred in 38.1% and 81.8% of patients in the two groups, with median PFS of 18.8 mon and 5.3 mon, respectively (*P* < 0.000, 1). Median OS was unreached in the combination group, and 27.8 mon in the monotherapy group (*P*=0.31). According to the *Cox* multivariate regression analysis, combination therapy was an independent prognostic factor of PFS.

**Conclusion:**

In patients with *EGFR*-mutant advanced lung adenocarcinoma with concomitant non-*EGFR* genetic alterations, combination of TKIs and chemotherapy was significantly superior to EGFR-TKIs monotherapy, which should be the preferred treatment option.

## Background

Lung cancer is the leading cause of cancer-related death worldwide^[1]^, and non-small cell lung cancer (NSCLC) accounts for approximately 85% of all cases^[2]^. NSCLC with sensitive epidermal growth factor receptor (*EGFR*) mutations may be susceptible to treatment with EGFR tyrosine kinase inhibitors (EGFR-TKIs), a breakthrough in lung cancer treatment this century that has opened a new chapter in the targeted therapy of solid tumors. At present, NSCLC with *EGFR* mutations has become the most important subtype of NSCLC. The *EGFR* mutation rate is as high as 51.4% in Asian patients with lung adenocarcinoma^[3]^, making it particularly important to optimize the treatment protocol for NSCLC with *EGFR* mutations. Ongoing in-depth research has raised new questions about treatment of NSCLC with *EGFR* mutations, the most important of which is the effect of combination therapy with EGFR-TKIs and other drugs, especially chemotherapy drugs.

The clinical trial NEJ009 has shown promising results of chemotherapy combined with a first-generation EGFR-TKIs: Among patients with sensitive *EGFR* mutations receiving Pemetrexed/Carboplatin combined with Gefitinib, progression-free survival (PFS) is 20.9 mon, and overall survival (OS) is 50.9 mon^[4]^, suggesting that combination therapy may be a potential new treatment protocol. However, the mechanism and the target population of combination therapy are unknown. One hypothesis is that for NSCLC patients with both *EGFR* mutation and concomitant non-*EGFR* genetic alterations, combination therapy inhibits the EGFR pathway and also counteracted the bypass activation associated with the concomitant alterations, thereby achieving better efficacy. To date, no evidence-based study is available to support this hypothesis. This study was designed to test this hypothesis.

## Materials and Methods

### Patients

We searched the electronic medical records of the Affiliated Cancer Hospital and the First Affiliated Hospital of Zhengzhou University to include patients treated between January 2018 and May 2020 who met the following criteria: histologically confirmed lung adenocarcinoma; clinical or pathological stage Ⅳ [tumor-node-metastasis (TNM) stage, edition 8]; performance status (PS) score 0-2; and next-generation sequencing with biopsy specimens at initial diagnosis. Due to the retrospective nature of this analysis, we were unable to ensure any consistent testing platform or panel. The panels all included at least *EGFR*, anaplastic lymphoma kinase (*ALK*), proto-oncogene tyrosine-protein kinase 1 (*ROS1*), Kirsten rat sarcoma virus gene (*KRAS*), c-Met tyrosine kinase gene (*MET*), rapidly accelerated fibrosarcoma (*RAF*), human epidermal growth factor receptor 2 (*HER2*), rearranged during transfection (*RET*), and tumor protein 53 (*TP53*); sensitive *EGFR* mutations (exons 18-21); and at least one non-*EGFR* mutation. The patients had to have first-line treatment with a first- to third-generation EGFR-TKIs alone or in combination with chemotherapy, at least one evaluable lesion [per Response Evaluation Criteria in Solid Tumors (RECIST) 1.1], and complete imaging data.

Based on first-line treatment mode, the patients were divided into the monotherapy (targeted therapy) group and the combination therapy (targeted therapy combined with chemotherapy) group. Information such as demographics, PS score, stage (IVA, IVB), central nervous system (CNS) metastases, *EGFR* mutation sites, type of concomitant non-*EGFR* alterations, and first-line treatment protocol was collected.

### Efficacy evaluation and follow-up

Imaging evaluation were performed every 6 weeks after the initial dosing, including enhanced chest and upper abdominal computed tomography (CT) and enhanced CT of any tumor site present at baseline, as well as enhanced brain magnetic resonance imaging (MRI) in patients with CNS metastases. Electronic imaging data were retrieved, and the efficacy was evaluated based on RECIST 1.1 to determine best response and the time of disease progression. The patients were followed up by face-to-face visit or telephone to collect their survival status. Endpoints included PFS, OS, objective response rate (ORR), and disease control rate (DCR). PFS was defined as the time from initial dosing to disease progression (per RECIST 1.1) or death. OS was defined as the time from initial dosing to death.

### Statistical analysis

The chi-squared test or *Fisher's* exact test was performed to compare ORR and DCR between the two groups. The *Kaplan-Meier* method was used for survival analysis and to plot PFS and OS curves. The *Log-rank* test was used to analyze the differences in PFS and OS between the two groups. *Cox* multivariate regression analysis was performed to determine if treatment protocol was an independent prognostic factor. Factors included in the *Cox* regression analysis were gender, age, PS score, stage, CNS metastases, *EGFR* mutation sites, non-*EGFR* mutations, and TKIs. We used GraphPad Prism 8.0 to perform survival analysis. And all other statistical analyses were performed by SPSS v25.0.

## Results

A total of 107 eligible patients were included in this study, including 63 cases in the combination group and 44 cases in the monotherapy group. Sixty-seven patients were women and 40 were men. The mean age was (58.3±12.1) years. PS score was 0-1 in all the patients except 2 patients in the monotherapy group (PS=2). *EGFR* mutations were all exon 19 deletion or exon 21 L858R point mutation, except for 1 rare mutation in each group (S768I and G719C).

As for concomitant alterations, 83 of 107 patients had a single mutation, including *TP53* mutation (*n*=53), *MET* amplification (*n*=13), *KRAS* mutation (*n*=3), and other mutations [*n*=14; including *BRAF* mutation, *HER2* amplification, cyclin dependent kinase 4 (*CDK4*) amplification, phosphatase and tensin homolog (*PTEN*) mutation, discoidin domain receptor tyrosine kinase 2 (*DDR2*) mutation, TSC complex subunit 1 (*TSC1*) mutation, and phosphatidylinositol-4, 5-bisphosphate 3-kinase catalytic subunit alpha (*PIK3CA*) mutation]. Twenty-four patients had two or more concomitant alterations, of whom 19 patients had *TP53* mutation combined with other alterations [including ataxia telangiectasia-mutated gene (*ATM*) mutation, SMAD family member 4 (*SMAD4*) mutation, *MET* amplification, *MYC* proto-oncogene (*MYC*) amplification, APC regulator of WNT signaling pathway (*APC*) mutation, *PIK3CA* mutation, catenin beta 1 (*CTNNB1*) mutation, neurotrophic receptor tyrosine kinase 1 (*NTRK1*) rearrangement, retinoblastoma 1 (*RB1*) mutation, AXL receptor tyrosine kinase (*AXL*) mutations, *ALK* mutations, and *CDK4* mutations]. See Table 1 for the balanced baseline characteristics between the two groups.

**Table 1:** Baseline demographics and clinical characteristics

Characteristics	Total (*n*=107)	Monotherapy (*n*=44)	Combination therapy (*n*=63)	*P*
Gender (Female/Male)	67/40	30/14	37/26	0.320
Age (yr)	58.3±12.1	59.2±12.8	57.7±11.7	0.681
ECOG PS				0.175
0	24	8	16	
1	81	34	47	
2	2	2	0	
Stage				0.049
IVA	22	5	17	
IVB	85	39	46	
*EGFR* mutation sites				0.950
Exon 19 deletion	50	20	30	
Exon 21 L858R	55	23	32	
Other	2	1	1	
Concomitant genetic alterations				0.044
*TP53* mutation	53	16	37	
*MET* amplification	13	10	3	
*KRAS* mutation	3	1	2	
Other single mutations	14	6	8	
≥2 mutations	24	11	13	
TKIs				0.150
First-generation	102	40	62	
Second-generation	2	2	0	
Third-generation	3	2	1	
ECOG: Eastern Cooperative Oncology Group; PS: performance status; EGFR: epidermal growth factor receptor; TKIs: tyrosine kinase inhibitors.				

First-generation TKIs were used in 62 of 63 patients in the combination group and 40 of 44 patients in the monotherapy group (only a few patients received second- or third-generation TKIs)(Tab 1). Chemotherapy in the combination group: 38 patients received Pemetrexed combined platinum, 17 patients received Pemetrexed and Platinum combined Bevacizumab, 6 patients received Pemetrexed alone, and 2 patients received a non-Pemetrexed platinum-based two-drug regimen. The median number of treatment cycles was 6 (1-32).

In the combination group, 49 patients achieved partial response (PR), 12 achieved stable disease (SD), and 2 achieved progressive disease (PD). In the monotherapy group, 22 patients achieved PR, 12 had SD, and 10 had PD. The ORR was 78% in the combination group and 50% in the monotherapy group (*P*=0.003), and the DCR was 97% and 77% respectively (*P*=0.002).

**Figure 1:**
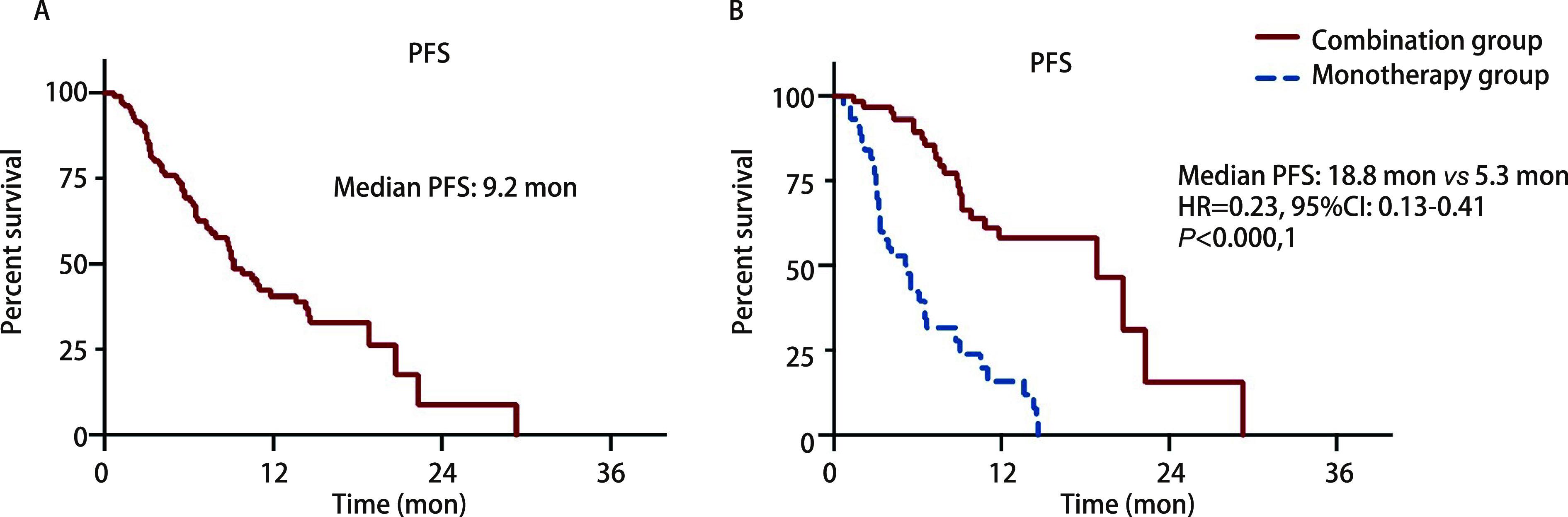
Progression-free survival (PFS) of all enrolled patients (A) and patients of combination group or monotherapy group (B)

The patients were followed up through August 24, 2020, with a median follow-up time of 13.7 mon. As of last follow-up, 60 patients occurred PFS events (56.1%), and the median PFS was 9.2 mon (Fig 1A). PFS events was observed in 24 patients (38.1%) in the combination group and 36 patients (81.8%) in the monotherapy group. Median PFS was 18.8 mon and 5.3 mon, respectively [hazard ratio (HR)=0.23; 95%CI: 0.13-0.41; *P* < 0.000, 1] (Fig 1B). Multivariate analysis showed that treatment protocol (combination therapy *vs* monotherapy) was an independent prognostic factor for PFS (HR=0.13; 95%CI: 0.06-0.28; *P* < 0.001). Gender and stage were also independent prognostic factors for PFS (Tab 2).

**Table 2:** MultivariateCoxregressionanalysis of prognostic factors on PFS of all enrolled patients

Variable	*n*	*P*	HR	95%CI
Gender		0.002	0.358	0.185-0.693
Female	67			
Male	40			
Age (yr)		0.201	0.585	0.275-1.330
> 65	30			
≤65	77			
ECOG PS		0.133	0.558	0.260-1.193
0	24			
1-2	83			
Clinical stage		0.003	0.404	0.221-0.737
IVA	22			
IVB	85			
CNS metastasis		0.330	0.717	0.367-1.400
Yes	39			
No	68			
*EGFR* mutation		0.297	1.393	0.747-2.596
Exon 19 deletion	50			
Other	57			
Concomitant non-*EGFR* mutation		0.611	0.841	0.431-1.640
*TP53*	53			
Other	54			
Number of concomitant non-*EGFR* mutations		0.152	0.478	0.174-1.313
1	83			
≥2	24			
Treatment protocol		< 0.001	0.132	0.062-0.281
Combination therapy	63			
TKIs monotherapy	44			
Type of EGFR-TKIs		0.182	0.275	0.041-1.831
First-generation	102			
Other	5			
CNS: central nervous system.				

**Figure 2:**
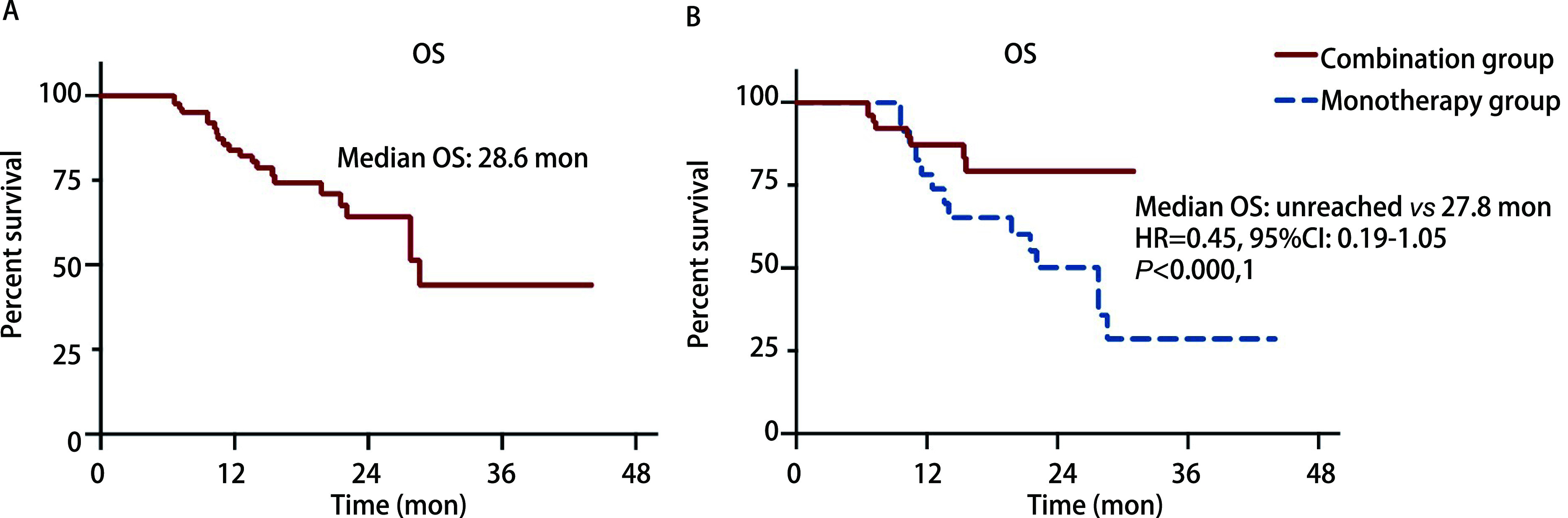
Overall survival (OS) of all enrolled patients (A) and patients of combination group or monotherapy group (B)

As of last follow-up, 22 patients (20.6%), including 8 patients (12.7%) in the combination group and 14 (31.8%) in the monotherapy group died, with a median OS of 28.6 mon (Fig 2A). The median OS had not been reached in the combination group, whereas the estimated median OS was 27.8 mon in the monotherapy group (HR=0.45; 95%CI: 0.19-1.05; *P*=0.31) (Fig 2B).

## Discussion

As soon as EGFR-TKIs became available, a series of clinical trials, such as INTACT 1^[5]^, INTACT 2^[6]^, TALENT^[7]^, and TRIBUTE^[8]^, were conducted to investigate the effect of chemotherapy alone or in combination with TKIs in non-selected patients with advanced NSCLC. However, the results from these studies were all negative. The reasons may include potential antagonism between platinum drugs and EGFR-TKIs^[9]^ or that cell cycle-specific chemotherapy drugs are difficult to play a role because EGFR-TKIs arrest the tumor cell cycle at G_1_ phase^[10]^.

Based on these hypotheses and the results from basic research, the combination strategy was largely eschewed for some time. However, in 2013, FASTACT-2, a large phase Ⅲ randomized controlled trial^[11]^, used a combination therapy protocol known as intercalated therapy to avoid concomitant use of chemotherapy and TKIs, thereby preventing cell cycle arrest from impairing the efficacy of chemotherapy. Specifically, a group of Asian patients with advanced NSCLC received 28-day cycles of chemotherapy with Gemcitabine (days 1, 8) and Carboplatin (day 1), as well as Erlotinib on days 15-28. After up to 6 cycles of chemotherapy, oral Erlotinib was given every day until disease progression. The results showed that PFS and OS were significantly longer in the chemotherapy-combined-with-Erlotinib group than in the Erlotinib-alone group. A similar study, ISCAN^[12]^, reached similar conclusions, although the time point for intercalated chemotherapy was slightly different.

In 2016, JMIT, a phase Ⅱ randomized controlled trial, was designed based on a different hypothesis, that platinum drugs and EGFR-TKIs are antagonistic^[13]^. Pemetrexed was given in combination with oral Gefitinib (daily, from day 1 of chemotherapy) without using an intercalated strategy. In addition, this was the first trial to enroll patients with advanced NSCLC and sensitive *EGFR* mutations. The results showed that PFS, the primary endpoint, was significantly longer in the combination group than in the Gefitinib-alone group.

In 2018, the initial results of study NEJ009^[14]^ challenged the presumed mechanism of the clinical benefits observed in FASTACT-2 and JMIT. The trial investigated the efficacy of Gefitinib alone or in combination with chemotherapy in patients with advanced non-squamous NSCLC and sensitive *EGFR* mutations. The chemotherapy regimen was Pemetrexed combined with Carboplatin, a platinum-based two-drug regimen, and Gefitinib was given from day 1 of chemotherapy without a preset interval. The trial achieved the best outcomes with chemo-targeted combination therapy, as this regime extended PFS from 11.9 mon to 20.9 mon (*P* < 0.001) and OS from 38.8 mon to 50.9 mon (*P*=0.021)^[4]^. In 2019, an Indian single center phase Ⅲ clinical trial with a similar design to NEJ009 produced similar results^[15]^. Results of these trials suggest that intercalated chemotherapy with TKIs (based on the theory of TKIs-induced cell cycle arrest) or the use of non-platinum-containing chemotherapy in combination with TKIs (based on the theory of antagonism between platinum drugs and TKIs) was unwarranted. The failure of early clinical trials is likely related to a lack of precise patient selection.

During this period, researchers are also developing a more in-depth understanding of lung cancer with *EGFR* mutations. High-throughput technology shows that 45.0% to 89.7% of patients with *EGFR* mutations also harbor a concomitant non-*EGFR* genetic alterations, and these patients are far less responsive to EGFR-TKIs than those with pure *EGFR* mutations^[16-18]^. This may be related to the resistance that rapidly develop in association with the activation of alternate pathways, and chemotherapy combined with TKIs may prevent rapid activation of alternate pathways because the regimen does not work solely by inhibiting the EGFR pathway. Our study indirectly confirms this hypothesis: In the TKIs monotherapy group, the ORR was 50%, the median PFS was 5.3 mon, which were significant lower or shorter than the historical data of first-line EGFR-TKIs therapies. In the combination group, the ORR was 78%, the median PFS was 18.8 mon, the HR of disease progression was reduced by 77%, and the HR of death was reduced by 55%. These data indicate that the combination therapy overcomes the shortcomings of TKIs monotherapy in patients with both *EGFR* mutations and concomitant non-*EGFR* genetic alterations, which may be one of the benefit logics of chemo-targeted combination strategy. Answering questions such as whether patients with a pure *EGFR* mutation will benefit from the combination therapy (and if so, what is the mechanism) and the clinical benefits relative to those observed in patients with both *EGFR* mutations and non-*EGFR* alterations will facilitate the precise selection of a treatment protocol.

This study has obvious limitations due to the nature of retrospective analyses and the small sample size. For example, this study showed that approximately 25% of patients harbored two or more non-*EGFR* mutations in this real-world clinical setting, but according to *Cox* multivariate regression analysis, the number of non-*EGFR* mutations was not an independent prognostic factor for PFS. The number of non-*EGFR* mutations seen in this study may be incorrect, or it may be unbalanced due to the different testing platforms and panels used across studies, which along with the small sample size makes it impossible to draw any definitive conclusion about the relationship between the number of non-*EGFR* mutations and the efficacy of combination therapy.

## Conclusion

The efficacy of combination therapy in patients with both *EGFR* and concomitant non-*EGFR* genetic alterations may be an important contributor to the superior efficacy of combination therapy over EGFR-TKIs monotherapy in patients with *EGFR* mutations in general. Combination of chemotherapy and EGFR-TKIs should be a priority treatment option for *EGFR* mutated patients with concomitant non-*EGFR* genetic alterations. However, given the nature of this retrospective analysis and the small sample size, prospective studies are needed to validate the results.

## Declarations

The American Journal Experts (AJE) provided English edit. All authors declared no actual or potential conflict of interest including any financial, personal or other relationships with other people or organizations within that could inappropriately influence (bias) this article.

This study was approved by the Ethics Committee of the Affiliated Cancer Hospital and the First Affiliated Hospital of Zhengzhou University. The approved number was 2020-329 and L2020-Y150, respectively. We confirm that all methods were performed in accordance with the relevant guidelines and regulations.

## References

[1] Sung H, Ferlay J, Siegel RL (2021). Global Cancer Statistics 2020: GLOBOCAN estimates of incidence and mortality worldwide for 36 cancers in 185 countries. CA Cancer J Clin.

[2] Herbst RS, Heymach JV, Lippman SM (2008). Lung cancer. N Engl J Med.

[3] Shi Y, Au JSK, Thongprasert S (2014). A prospective, molecular epidemiology study of *EGFR* mutations in Asian patients with advanced non-small-cell lung cancer of adenocarcinoma histology (PIONEER). J Thorac Oncol.

[4] Hosomi Y, Morita S, Sugawara S (2020). Gefitinib alone versus Gefitinib plus chemotherapy for non-small-cell lung cancer with mutated epidermal growth factor receptor: NEJ009 study. J Clin Oncol.

[5] Giaccone G, Herbst RS, Manegold C (2004). Gefitinib in combination with gemcitabine and cisplatin in advanced non-small-cell lung cancer: a phase Ⅲ trial--INTACT 1. J Clin Oncol.

[6] Herbst RS, Giaccone G, Schiller JH (2004). Gefitinib in combination with paclitaxel and carboplatin in advanced non-small-cell lung cancer: a phase Ⅲ trial--INTACT 2. J Clin Oncol.

[7] Gatzemeier U, Pluzanska A, Szczesna A (2007). Phase Ⅲ study of erlotinib in combination with cisplatin and gemcitabine in advanced non-small-cell lung cancer: the Tarceva Lung Cancer Investigation Trial. J Clin Oncol.

[8] Herbst RS, Prager D, Hermann R (2005). TRIBUTE: a phase Ⅲ trial of erlotinib hydrochloride (OSI-774) combined with carboplatin and paclitaxel chemotherapy in advanced non-small-cell lung cancer. J Clin Oncol.

[9] Tsai CM, Chen JT, Stewart DJ (2011). Antagonism between gefitinib and cisplatin in non-small cell lung cancer cells: why randomized trials failed?. J Thorac Oncol.

[10] Cheng H, An SJ, Zhang XC (2011). *In vitro* sequence-dependent synergism between paclitaxel and gefitinib in human lung cancer cell lines. Cancer Chemother Pharmacol.

[11] Wu YL, Lee JS, Thongprasert S (2013). Intercalated combination of chemotherapy and erlotinib for patients with advanced stage non-small-cell lung cancer (FASTACT-2): a randomised, double-blind trial. Lancet Oncol.

[12] Jian H, Li W, Ma Z (2017). Intercalating and maintenance gefitinib plus chemotherapy versus chemotherapy alone in selected advanced non-small cell lung cancer with unknown EGFR status. Sci Rep.

[13] Cheng Y, Murakami H, Yang PC (2016). Randomized phase Ⅱ trial of gefitinib with and without pemetrexed as first-line therapy in patients with advanced nonsquamous non-small-cell lung cancer with activating epidermal growth factor receptor mutations. J Clin Oncol.

[14] Nakamura A, Inoue A, Morita S (2018). Phase Ⅲ study comparing gefitinib monotherapy (G) to combination therapy with gefitinib, carboplatin, and pemetrexed (GCP) for untreated patients (pts) with advanced non-small cell lung cancer (NSCLC) with *EGFR* mutations (NEJ009). J Clin Oncol.

[15] Noronha V, Patil VM, Joshi A (2020). Gefitinib versus gefitinib plus pemetrexed and carboplatin chemotherapy in *EGFR*-mutated lung cancer. J Clin Oncol.

[16] Li XM, Li WF, Lin JT (2021). Predictive and prognostic potential of TP53 in patients with advanced non-small-cell lung cancer treated with EGFR-TKI: Analysis of a phase Ⅲ randomized clinical trial (CTONG 0901). Clin Lung Cancer.

[17] Zhao J, Bai H, Wang X (2022). Biomarker subset analysis of a phase Ⅲb, open-label study of afatinib in EGFR tyrosine kinase inhibitor-naive patients with *EGFR* m+ non-small-cell lung cancer. Future Oncol.

[18] Hong S, Gao F, Fu S (2018). Concomitant genetic alterations with response to treatment and epidermal growth factor receptor tyrosine kinase inhibitors in patients with *EGFR*-mutant advanced non-small cell lung cancer. JAMA Oncol.

